# Supplementary material for: Comparison of climatic factors on mosquito abundance at US Army Garrison Humphreys, Republic of Korea
Source: PLoS One. 2020 Oct 21;15(10):e0240363. doi: 10.1371/journal.pone.0240363 (PMC7577452; doi:10.1371/journal.pone.0240363)
Supplement: Attachment — Submitted filename: 2020-08-06 - Manuscript_PLOS ONE_Reviewers comment_response.docx [file pone.0240363.s001.docx]

**Reviewers' comments:**

Reviewer's Responses to Questions

Comments to the Author

**1. Is the manuscript technically sound, and do the data support the conclusions?**

The manuscript must describe a technically sound piece of scientific research with data that supports the conclusions. Experiments must have been conducted rigorously, with appropriate controls, replication, and sample sizes. The conclusions must be drawn appropriately based on the data presented.

Reviewer #1: Partly

Reviewer #2: Yes

**2. Has the statistical analysis been performed appropriately and rigorously?**

Reviewer #1: Yes

Reviewer #2: Yes

**3. Have the authors made all data underlying the findings in their manuscript fully available?**

The PLOS Data policy requires authors to make all data underlying the findings described in their manuscript fully available without restriction, with rare exception (please refer to the Data Availability Statement in the manuscript PDF file). The data should be provided as part of the manuscript or its supporting information, or deposited to a public repository. For example, in addition to summary statistics, the data points behind means, medians and variance measures should be available. If there are restrictions on publicly sharing data—e.g. participant privacy or use of data from a third party—those must be specified.

Reviewer #1: No

Reviewer #2: Yes

**4. Is the manuscript presented in an intelligible fashion and written in standard English?**

PLOS ONE does not copyedit accepted manuscripts, so the language in submitted articles must be clear, correct, and unambiguous. Any typographical or grammatical errors should be corrected at revision, so please note any specific errors here.

Reviewer #1: Yes

Reviewer #2: Yes

**5. Review Comments to the Author**

Please use the space provided to explain your answers to the questions above. You may also include additional comments for the author, including concerns about dual publication, research ethics, or publication ethics. (Please upload your review as an attachment if it exceeds 20,000 characters)

**Reviewer #1**

The authors collected a valuable dataset containing mosquito diversity and abundance information from an army base in the Republic of Korea (May-Oct 2018, May-Oct 2019). Mean daily temperature and daily precipitation data were obtained from a nearby weather station, and empirical mean temperature ranges for maximum abundance were determined for each mosquito species.

🡪 The authors deeply appreciate a comprehensive review of the manuscript by the reviewer and for the valuable comments and suggestions. We have responded to the comments and the manuscript was revised accordingly.

Again, the dataset is large and unique, but I do have some concerns regarding the analysis:

**Major comments**

1. A time lag of 2 weeks was included in the model. However, do larval development times vary per species? And did you consider the potential age range of the adult population? A 2-week time lag assumes you only collect very young adults in the traps. How would the temperature-abundance relation look if different time lags were included (which ideally take into account the larval development times and mean/median age of the adult population for each species, if you can find literature on that)?

🡪 We agree the reviewer’s comments. In general, the larval life cycle is known to be 10-14 days. Therefore, a lag of 2-weeks was applied based on the meteorological factors and in consideration of the adult period of young mosquitoes, which are generally known.

However, it also has been reported in a previous study that the most active period of mosquito adult activity was about 12 days (Burkett DA, Lee WJ, Lee KW, Kim HC, Lee HI, Lee JS, et al. Light, carbon dioxide, and octenol-baited mosquito trap and host-seeking activity evaluations for mosquitoes in a malarious area of the Republic of Korea. J Am Mosq Control Assoc. 2001;17:196-205).

In previous studies, they did not evaluate differences in the growth period of larvae for different mosquito species. In adult mosquitoes, the survival period of male adults is 2-3 weeks. For females, the oviposition cycle is about 3-4 days after a blood meal with overall survival of 4-5 weeks. Although mosquitoes were not classified by sex in this study, considering this period, the adult growth period was assumed to be the median period (average period) in this study (1-3 weeks for adult survival). The results of applying the temperature and cumulative precipitation for a lag of 3 to 5 weeks were compared (see Figures 1, 2, 3, below).

Figure 1. Relationship between the abundance of selected mosquito species and daily mean temperatures by generalized additive model (GAM), adjusted for the cumulative precipitation based on a 3-week lag effect during 2018 and 2019. The solid line is the estimated log (relative risk) as density of mosquitoes by species and dashed line is the 95% confidence interval. * Adjusted for a 3-week lag for cumulative precipitation, year, season, and application of insecticide by ULV fogging. RR, relative risk.

Figure 2. Relationship between the abundance of selected mosquito species and daily mean temperatures by generalized additive model (GAM), adjusted for the cumulative precipitation based on a 4-week lag effect during 2018 and 2019. The solid line is the estimated log (relative risk) as density of mosquitoes by species and dashed line is the 95% confidence interval. * Adjusted for a 4-week lag for cumulative precipitation, year, season, and application of insecticide by ULV fogging. RR, relative risk.

Figure 3. Relationship between the abundance of selected mosquito species and daily mean temperatures by generalized additive model (GAM), adjusted for the cumulative precipitation based on a 5-week lag effect during 2018 and 2019. The solid line is the estimated log (relative risk) as density of mosquitoes by species and dashed line is the 95% confidence interval. * Adjusted for a 5-week lag for cumulative precipitation, year, season, and application of insecticide by ULV fogging. RR, relative risk.

Compared with the results presented in the text, the range of the maximum abundance of mosquitoes by species after the adjustment of cumulative precipitation in the expanded lag was similar. However, as the range of lag expanded (lag 4, 5-weeks), the abundance of *Anopheles* Hyrcanus group and *Culex pipiens* showed a decrease as the temperature increased. Due to these effects, the relationship between the temperature of lag 4 and 5 weeks and total mosquito abundance was not statistically significant.

In the previous results, when a lag of 2-weeks was applied, the relationship between relative mosquito abundance and temperature, adjusted for cumulative precipitation, was clearly shown. Therefore, we applied the 2-week lag for temperature.

The strength of this study was the consecutive daily collection of mosquitoes conducted three days/week, so the numbers of collected mosquitoes, by species, were counted daily and not an average of 3-days. Most studies only collect mosquitoes once a week and analyzed data by matching the cumulative number of mosquitoes and meteorological indices per week. Therefore, we believe that our study has less limitation in deriving an accurate dose-response relationship between the temperature and the abundance of mosquitoes.

2. On the topic of ‘time lags’: adult control applied with a ULV fogger was adjusted for all models, looking at a period of 1-4 days post-intervention. I am not familiar with the study area, but given the description it may be impossible to see an effect, given the amount of breeding sites (and thus mosquitoes) surrounding the army base. In addition, an effect is expected (i) after the next gonotrophic cycle has been completed, and not the next day, for many species, and (ii) 1-2 weeks later given the reduced numbers of eggs/larvae that go into the system. This makes me wonder if the current analysis is valid (to conclude ULV is not effective).

🡪 The time lag was based on previous studies, e.g., Foley et al. (2012) who demonstrated a bi-modal distribution of *Anopheles* spp. in the ROK that corresponded with increased numbers of malaria positive mosquitoes. Masuoka et al. (2010) showed that as minimum temperatures increased in July, so did the populations of *Culex tritaeniorhynchus*. Additionally, Richards et al. (2010) showed a close relationship to rice paddies, which are adjacent to Camp Humphreys. However, these data were based on population numbers, not adult survival.

After taking the comments into account, the authors reanalyzed the effectiveness of the ULV fogger. Existing results analyzed the mosquito abundance applied to the delay effect (lag effect) of ULV fogging over the entire collection period, but this could be considered to be meaningless, according to the reviewer’s comment.

For the reanalysis, we established two strategies. First, we compared the mosquito abundance of the day before and after the ULV fogging. Second, according to the reviewer’s comment, the ULV fogger was an anti-adult agent, but it was assumed that this could affect the larvae’s population. Therefore, we observed a decrease in mosquito abundance by applying a delay effect of 1 and 2 weeks.

First, the abundance of mosquito on the day before and after the ULV fogging was compared by species (see Figure 4). Overall, the number of mosquitoes collected decreased on the following day compared to the day before the fogging and it was statistically significant (*p*<0.05). In the results classified by species, numbers of *Aedes albopictus, Aedes lineatopennis, Culex pipiens*, and *Culex bitaeniorhynchus* collected decreased significantly. For other species, the numbers of mosquitoes collected decreased the day after fogging, but the decrease in number was not statistically significant.

 It is beneficial to understand that reductions in collected numbers depended on mosquito species, but that significant decreases in numbers collected were not observed for all species. The results for selected species are presented in Figure 4 of the manuscript.

Figure 4. Comparison the mean numbers (range) of mosquitoes collected daily using Mosquito Magnet® Independence model® traps operated at 12 collection sites at USAG Humphreys 1-day before and after pesticide application using a ULV fogger during 2018. *Adjusted for a lag of 2-weeks for daily mean temperatures and cumulative precipitation, season, RR. Relative risk; CI, confidence interval.

To compare the residual effects of the day before and after ULV fogging, the numbers of mosquitoes collected at 1-week and 2-week were estimated by species. Overall, while the relative abundance of mosquitoes decreased after 1-week, the decrease was not statistically significant (see Table 1). Additionally, after 2-week, there was no residual effect of ULV fogging, but the numbers of mosquitoes collected increased (see Table 2). This indicates that ULV fogging has a minor temporary effect on reduction of adult mosquitoes.

Table 1. Comparison the mean numbers (range) of mosquitoes collected daily using Mosquito Magnet® Independence model® traps operated at 12 collection sites at USAG Humphreys 1 day before and 1 week after pesticide application using a ULV fogger during 2018.

| **Species** | **Exposure** | **Log (RR)^*^** | **95% CI** | ***p*-value** |
| --- | --- | --- | --- | --- |
| Total | Before 1 day | ref |  |  |
|  | After 1 week | -0.13 | (-0.60 - 0.34) | 0.63 |
| *Aedes albopictus* | Before 1 day | ref |  |  |
|  | After 1 week | 0.97 | (0.56 - 1.39) | 0.05 |
| *Aedes lineatopennis* | Before 1 day | ref |  |  |
|  | After 1 week | 0.49 | (-0.36 - 1.35) | 0.92 |
| *Aedes vexans nipponii* | Before 1 day | ref |  |  |
|  | After 1 week | 0.6 | (-0.24 - 1.44) | 0.30 |
| *Anopheles* Hyrcanus Group^a^ | Before 1 day | ref |  |  |
|  | After 1 week | 0.58 | (-1.19 - 2.35) | 0.59 |
| *Culex pipiens* | Before 1 day | ref |  |  |
|  | After 1 week | -0.47 | (-1.26 - 0.30) | 0.36 |
| *Culex orientalis* | Before 1 day | ref |  |  |
|  | After 1 week | 0.2 | (-0.47 - 0.87) | 0.64 |
| *Culex bitaeniorhynchus* | Before 1 day | ref |  |  |
|  | After 1 week | -0.04 | (-1.23 - 1.15) | 0.95 |
| *Culex tritaeniorhynchus* | Before 1 day | ref |  |  |
|  | After 1 week | -0.26 | (-0.68 - 0.16) | 0.34 |
| *Mansonia uniformis* | Before 1 day | ref |  |  |
|  | After 1 week | 1.04 | (0.76 - 1.32) | 0.04 |

*Adjusted for a lag of 2-weeks for daily mean temperature and cumulative precipitation, season, RR. Relative risk; CI, confidence interval. ^a^*Anopheles* Hyrcanus Group includes *An. sinensis s.s., An. belenrae, An. lesteri, An. kleini*, and *An. pullus* that cannot be identified by morphological techniques.

Table 2. Comparison the mean numbers (range) of mosquitoes collected daily using Mosquito Magnet® Independence model® traps operated at 12 collection sites at USAG Humphreys 1 day before and 2 weeks after pesticide application using a ULV fogger during 2018.

| **Species** | **Exposure** | **Log (RR)^*^** | **95% CI** | ***p*-value** |
| --- | --- | --- | --- | --- |
| Total | Before 1 day | ref |  |  |
|  | After 2-week | 0.25 | (-0.15 - 0.65) | 0.44 |
| *Aedes albopictus* | Before 1 day | ref |  |  |
|  | After 2-week | 0.37 | (-0.05 - 0.79) | 0.69 |
| *Aedes lineatopennis* | Before 1 day | ref |  |  |
|  | After 2-week | 0.36 | (-0.36 - 1.35) | 0.88 |
| *Aedes vexans nipponii* | Before 1 day | ref |  |  |
|  | After 2-week | 2.88 | (-0.24 - 1.44) | 0.28 |
| *Anopheles* Hyrcanus Group^a^ | Before 1 day | ref |  |  |
|  | After 2-week | 1.41 | (-1.19 - 2.35) | 0.21 |
| *Culex pipiens* | Before 1 day | ref |  |  |
|  | After 2-week | 0.36 | (-1.26 - 0.30) | 0.87 |
| *Culex orientalis* | Before 1 day | ref |  |  |
|  | After 2-week | -0.13 | (-0.47 - 0.87) | 0.78 |
| *Culex bitaeniorhynchus* | Before 1 day | ref |  |  |
|  | After 2-week | 2.72 | (-1.23 - 1.15) | 0.19 |
| *Culex tritaeniorhynchus* | Before 1 day | ref |  |  |
|  | After 2-week | 0.58 | (-0.68 - 0.16) | 0.62 |
| *Mansonia uniformis* | Before 1 day | ref |  |  |
|  | After 2-week | 0.59 | (0.76 - 1.32) | 0.03 |

*Adjusted for a lag of 2-weeks for daily mean temperatures and cumulative precipitation, season, RR. Relative risk; CI, confidence interval. ^a^*Anopheles* Hyrcanus Group includes *An. sinensis s.s., An. belenrae, An. lesteri, An. kleini*, and *An. pullus* that cannot be identified by morphological techniques.

3. Line 342 “Third, there is a limitation that classification by sex of mosquitoes was not possible according to the mosquito species, since the Mosquito Magnet® infrequently collects male mosquitoes”. I do not fully understand this sentence. Why was it not possible to distinguish the males from the females? Males have more fine hairs on their antennae, correct? This sentence came a bit ‘out of the blue’ for me, as I assumed you were showing results for the females, as they are relevant for disease transmission. How does this affect your results?

🡪 This sentence was modified and the discussion that Mosquito Magnets infrequently collect males was deleted, since this did not pertain to the study. Males and females are easily identified, it is just that Mosquito Magnets collect mostly host-seeking mosquitoes and infrequently collects males, as occurs with New Jersey light traps.

4. In the discussion I am missing a section on climatic data (such as extremes, seasonal and daily fluctuations, micro-climatic conditions) and mosquito behaviors (thermal avoidance, resting biology).

🡪 As a reviewer’s comment, we have put this in the discussion section.

**Minor comments**

1. Why the May-Oct periods? Is this a particular season?

🡪 Mosquitoes are present as early as April at Camp Humphreys, but in very low numbers. Thus, adult surveillance begins the first week of May.

2. Line 96, Remove ‘e.g.’

🡪 Following the reviewer’s comment, we deleted this word in line 96.

3. Line 236, “the maximum daily mean precipitation for each of the years (2018, 124.8 mm; 2019, 108.0 mm) were similar” How are these similar?

🡪 While the duration and overall precipitation was much greater (4.1 X) during 2019, the daily maxims were similar, that is, the daily rainfall did not result in increased flooding caused by intense rainfall over a short period.

**Reviewer #2**

The manuscript entitled “Comparison of climatic factors on mosquito abundance at US Army Garrison Humphreys, Republic of Korea” analyzed the effect of climate conditions for different mosquito species. The study design and analysis are sound and appropriate. However, what kind of benefit can be delivered to vector control or disease prevention from the finding? The differences of maximum abundance temperature of each species are quite close.

🡪 The authors deeply appreciate a comprehensive review of the manuscript by the reviewer and for the valuable comments and suggestions. The manuscript was revised accordingly.

**Major comments**

1. The figure should demonstrate the landscape of different traps however, the white area was masked this information. It should be corrected. I understand it’s a military territory. If you are not allowed to show the landscape, at least describe the ecological feature for each trap.

🡪 We agree that the reviewer comments are important. However, this area is a military area of the United States, not the jurisdiction of the Republic of Korea, so a copy of the current map not supposed to be provided.

2. The lag effects of temperature and precipitation on mosquito abundance have been discussed in many papers. Why do you decide to evaluate the effects from 7-13 days prior the collection only?

🡪 Reviewer’s comments are very important. In general, the larval life cycle is known as 10-14 days. Therefore, in this study, the meteorological factors for a lag of 2-weeks was applied in consideration of the adult period of young mosquitoes, which are generally known.

However, it has also been reported in the previous study that the most active period of mosquito adult activity is about 12 days (Burkett DA, Lee WJ, Lee KW, Kim HC, Lee HI, Lee JS, et al. Light, carbon dioxide, and octenol-baited mosquito trap and host-seeking activity evaluations for mosquitoes in a malarious area of the Republic of Korea. J Am Mosq Control Assoc. 2001;17:196-205).

Also, in previous studies, they did not evaluate differences in the growth period of larvae for different mosquito species. For adult mosquitoes, the survival period of male adults is 2-3 weeks. For females, the oviposition cycle is about 3-4 days after a blood meal and survival of 4-5 weeks. Although mosquitoes were not classified by sex in this study, considering this period, the adult growth period was assumed to be the median period (average period) in this study (1-3 weeks for adult survival). The results of applying temperature and cumulative precipitation of for a lag 3 to 5 weeks were compared (see Figures 1, 2, 3, above). For a lag of 4 and 5 weeks, see comments above. Based on previous results, a lag of 2-weeks was applied (see comment above). Strengths of the study are also discussed above.

3. I suggest show the spatial distributions of mosquito abundance as a figure (map) for better presentation. The abundance of different species in the two years are significantly different.

🡪 We agree with the reviewer’s comment. In Figure 2 (in the manuscript), we mapped the abundance distribution by year as a proportion of the number of mosquito species/total number of mosquito populations collected. The proportion was mapped to 12 sites for selected species using the ArcGIS software. This was visualized to clearly show that the percentage collected by year is different for some species.

Figure 5. Distribution of the mean numbers of mosquitoes collected, by species, at USAG Humphreys, Pyeongtaek, Republic of Korea using Mosquito Magnet® Independence® model traps during 2018 and 2019. *Ratio was calculated as the number of mosquitoes collected by species/the number of total mosquitoes.

4. Regarding the effect of ULV in Figure3, do you comparing mosquito abundances to one-day before the ULV applied? You have mentioned that mosquito samples were collected three-day a week, how do you compare the effect of ULV in a consecutive 4 days?

🡪 After taking the comments into account, the authors reanalyzed the effectiveness of the ULV fogger (See comments and figures above).

**6. PLOS authors have the option to publish the peer review history of their article (what does this mean?). If published, this will include your full peer review and any attached files.**

If you choose “no”, your identity will remain anonymous but your review may still be made public.

Do you want your identity to be public for this peer review? For information about this choice, including consent withdrawal, please see our Privacy Policy.

Reviewer #1: No

Reviewer #2: No
